# Indoxyl Sulfate Promotes Macrophage IL-1β Production by Activating Aryl Hydrocarbon Receptor/NF-κ/MAPK Cascades, but the NLRP3 inflammasome Was Not Activated

**DOI:** 10.3390/toxins10030124

**Published:** 2018-03-15

**Authors:** Takuya Wakamatsu, Suguru Yamamoto, Toru Ito, Yoko Sato, Koji Matsuo, Yoshimitsu Takahashi, Yoshikatsu Kaneko, Shin Goto, Junichiro James Kazama, Fumitake Gejyo, Ichiei Narita

**Affiliations:** 1 Division of Clinical Nephrology and Rheumatology, Niigata University Graduate School of Medical and Dental Sciences, Niigata 951-8510, Japan; tkywakamatsu@yahoo.co.jp (T.W.); itotoru.gt@gmail.com (T.I.); yokosato0415@hotmail.com (Y.S.); ko.matsu.notre@gmail.com (K.M.); kanekoy@med.niigata-u.ac.jp (Y.K.); gotos@med.niigata-u.ac.jp (S.G.); naritai@med.niigata-u.ac.jp (I.N.); 2 Department of Clinical Engineering and Medical Technology Faculty Medical Technology, Niigata University of Health and Welfare, Niigata 950-3102, Japan; yoshimitsu2003@gmail.com; 3 Department of Nephrology and Hypertension, Fukushima Medical University, Fukushima 960-1247, Japan; jjkaz@fmu.ac.jp; 4 Niigata University of Pharmacy and Applied Life Sciences, Niigata 956-0841, Japan; gejyo@med.niigata-u.ac.jp

**Keywords:** uremic toxins, indoxyl sulfate, macrophage, aryl hydrocarbon receptor, nuclear factor-κB, inflammasome, atherosclerosis, cardiovascular disease

## Abstract

In chronic kidney disease (CKD) patients, accumulation of uremic toxins is associated with cardiovascular risk and mortality. One of the hallmarks of kidney disease-related cardiovascular disease is intravascular macrophage inflammation, but the mechanism of the reaction with these toxins is not completely understood. Macrophages differentiated from THP-1 cells were exposed to indoxyl sulfate (IS), a representative uremic toxin, and changes in inflammatory cytokine production and intracellular signaling molecules including interleukin (IL)-1, aryl hydrocarbon receptor (AhR), nuclear factor (NF)-κ, and mitogen-activated protein kinase (MAPK) cascades as well as the NLRP3 inflammasome were quantified by real-time PCR, Western blot analysis, and enzyme-linked immunosorbent assay. IS induced macrophage pro-IL-1β mRNA expression, although mature IL-1 was only slightly increased. IS increased AhR and the AhR-related mRNA expression; this change was suppressed by administration of proteasome inhibitor. IS promoted phosphorylation of NF-κB p65 and MAPK enzymes; the reaction and IL-1 expression were inhibited by BAY11-7082, an inhibitor of NF-κB. In contrast, IS decreased NLRP3 and did not change ASC, pro-caspase 1, or caspase-1 activation. IS-inducing inflammation in macrophages results from accelerating AhR-NF-κB/MAPK cascades, but the NLRP3 inflammasome was not activated. These reactions may restrict mature IL-1β production, which may explain sustained chronic inflammation in CKD patients.

## 1. Introduction

Chronic kidney disease (CKD) patients have a higher mortality risk than the general population, and is related to a higher incidence of cardiovascular disease (CVD) [1]. In addition to traditional risk factors for CVD, some CKD-specific risk factors are related to the development of CVD in patients with CKD [2]. Accumulation of protein-bound uremic toxins (PBUTs) in atherosclerotic lesions is an important factor in CKD-related CVD. PBUTs are increased as kidney function deteriorates and are difficult to eliminate by conventional dialysis treatments because of their protein-binding abilities [3]. For example, the serum level of indoxyl sulfate (IS), a representative PBUT, increases with CKD progression, and a high serum IS level is associated with a higher incidence of cardiovascular mortality [4]. In a mouse model, kidney damage accelerated atherosclerosis with increased IS deposition and inflammatory cytokine expression in the lesions; reduction of IS deposition in the lesion with oral charcoal adsorbent inhibited the acceleration of atherosclerosis [5]. Thus, IS may directly react with cells within atherosclerotic lesions.

Macrophage foam cell formation is a hallmark of atherosclerosis acceleration. In CKD, the pro-inflammatory M1 phenotype of macrophages becomes more apparent, while the anti-inflammatory M2 phenotype is less apparent [6,7]. We previously showed that IS directly reacts with macrophages and induces the production of reactive oxygen species and pro-inflammatory cytokines, including interleukin 1 (IL-1 and tumor necrotic factor and impairs cholesterol efflux to high-density lipoprotein [8].

IS induces various cellular inflammatory reactions through the aryl hydrocarbon receptor (AhR), mitogen-activated protein kinase (MAPK) (JNK), nuclear factor (NF)-κ, and the inflammasome [9,10,11,12,13]; however, no studies have investigated the detailed mechanisms of IS-induced macrophage inflammation.

In this study, we examined the roles of AhR/NF-κB/MAPK and the NLRP3 inflammasome in macrophages reacted with IS in vitro.

## 2. Results

### 2.1. Indoxyl Sulfate Enhanced Production of IL-1β Reacting with Aryl Hydrocarbon Receptor in Macrophages

**Figure 1:**
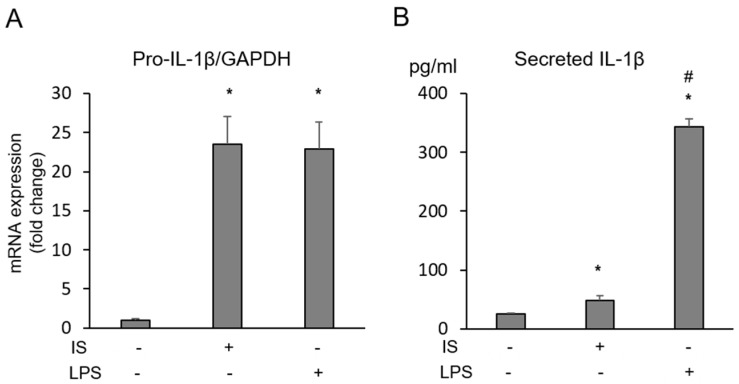
Production of IL-1β in macrophages exposed to IS (1 mM, 213 μg/mL) or LPS (100 ng/mL) for 24 h. Pro-IL-1β expression and mature IL-1 production caused by IS on THP-1 macrophage were examined by real-time PCR (**A**) and ELISA (**B**), respectively. Data represent the mean ± SD of three experiments. * *p* < 0.05 vs. control (without IS and LPS), and # *p* < 0.05 vs. with IS.

**Figure 2:**
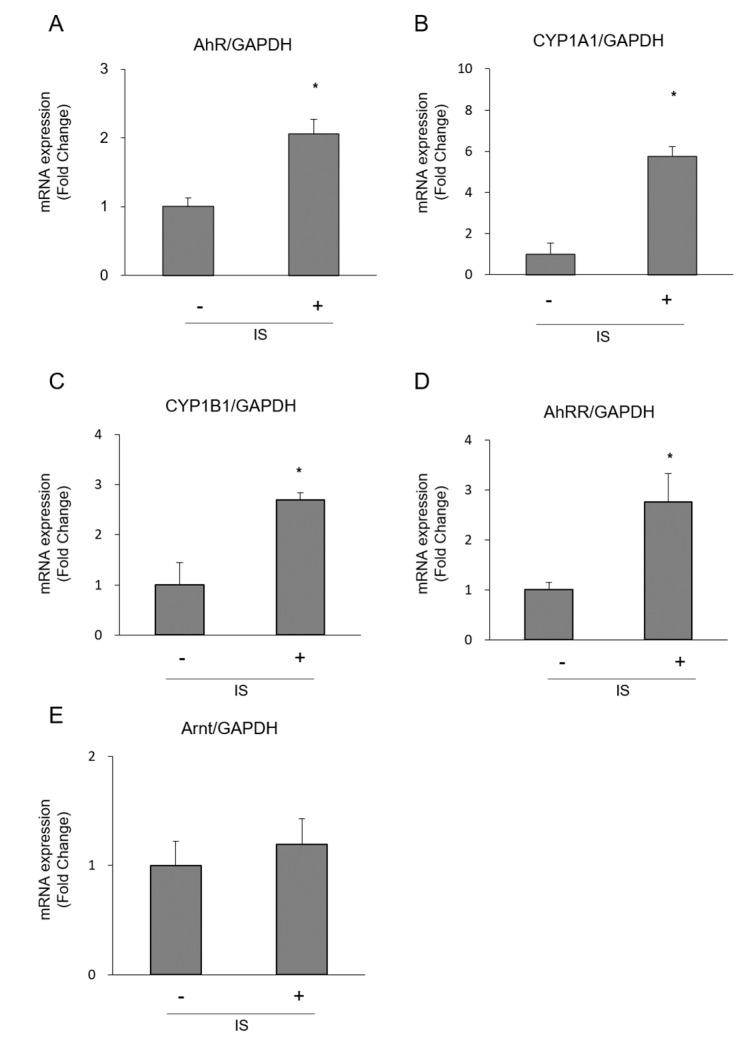
Activation of AhR pathway in THP-1 macrophages exposed to IS. (**A**) AhR; (**B**) CYP1A1; (**C**) CYP1B1; (**D**) AhRR; and (**E**) Arnt expression were determined by real-time PCR. Data represent the mean ± SD of three experiments. * *p* < 0.05 vs. without IS.

**Figure 3:**
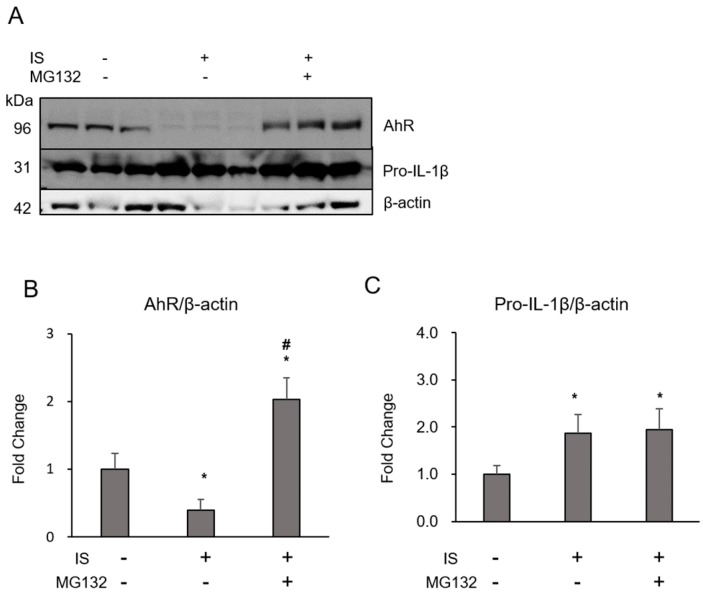
Effect of 1 mM IS and 10 µM MG132 on intracellular AhR and pro-IL-1β was determined by (**A**) Western blotting and quantification (**B**: AhR/-actin, **C**: pro-IL-1/-actin). Data represent the mean ± SD of three experiments. * *p* < 0.05 vs. without IS.

IS (1 mM, 213 μg/mL) enhanced macrophage pro-IL-1 mRNA expression (Figure 1A) and the production of mature IL-1 (Figure 1B). In this reaction, the increase in pro-IL-1 expression appeared to be more obvious than the increase in mature IL-1 production when compared to the reaction with lipopolysaccharide (LPS, 100 ng/mL) in macrophages. IS increased the mRNA expression of AhR, aryl hydrocarbon receptor repressor (AhRR), cytochrome P450 1A1 (CYP1A1), and cytochrome P450 1B1 (CYP1B1) significantly, and only showed trend to increase aryl hydrocarbon receptor nuclear translocator (Arnt), which are involved in the classical activation cascade of AhR (Figure 2). MG132, a proteasome inhibitor, recovered IS-induced reduction of AhR expression, but it did not inhibit pro-IL-1 expression induced by IS (Figure 3).

### 2.2. Indoxyl Sulfate Activated NF-κB/MAPK Signaling Pathways in Macrophages

**Figure 4:**
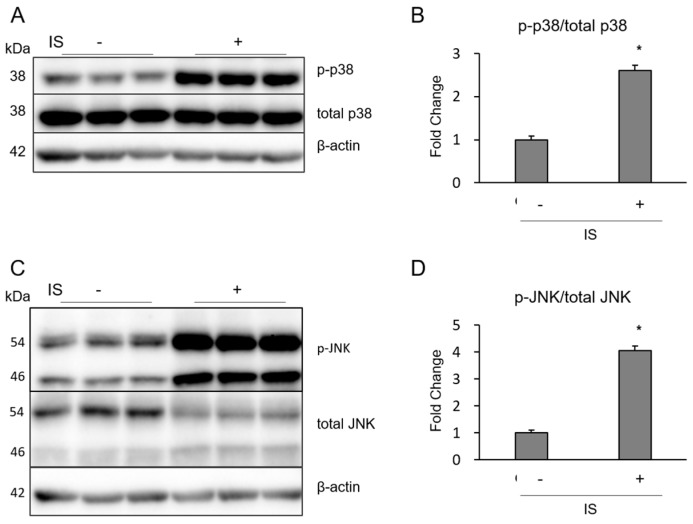

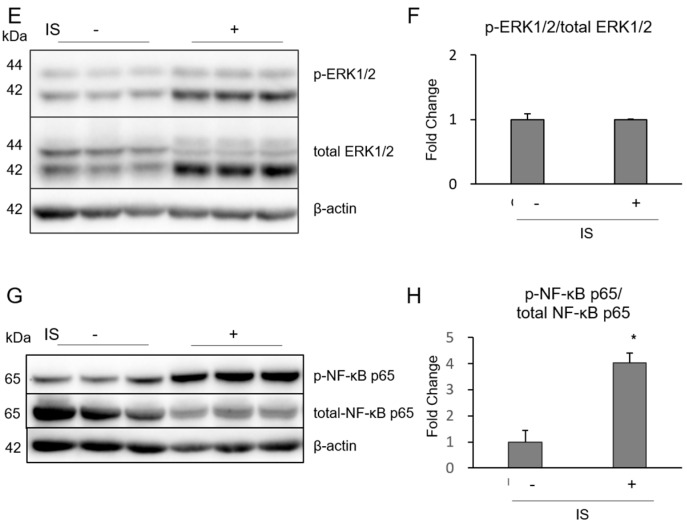
Phosphorylation of MAPK/NF-κB in THP-1 macrophages exposed to IS. Effect of IS on (**A**,**B**) p38; (**C**,**D**) JNK; (**E**,**F**) ERK1/2; and (**G**,**H**) NF-κB p65 phosphorylation were determined by Western blotting. Data represent the mean ± SD of three experiments. * *p* < 0.05 vs. without IS.

**Figure 5:**
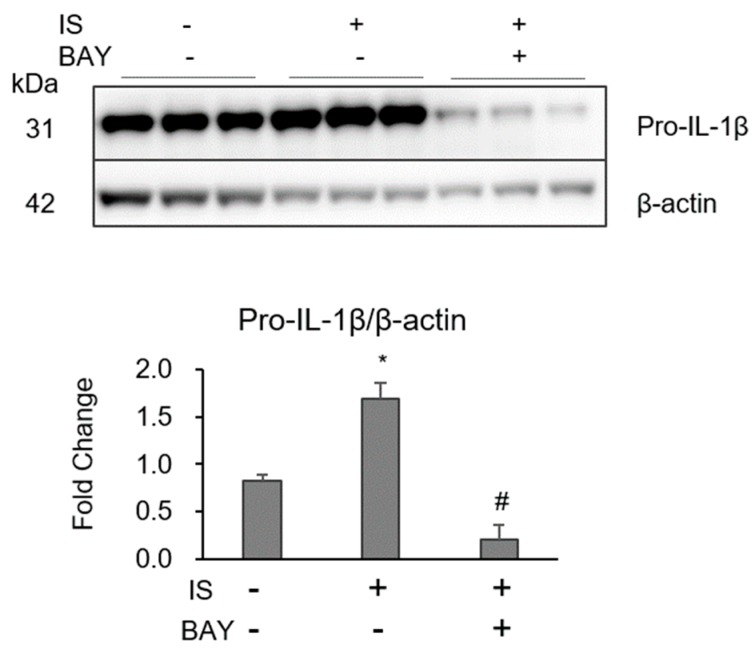
Effect of NF-κB inhibitor on pro IL-1 expression in THP-1 macrophages expose to IS. Effect of 1 mM IS and 10 µM BAY11-7082 on pro-IL-1β were determined by Western blotting. Data represent the mean ± SD of three experiments. * *p* <0.05 vs. without IS and BAY, and # *p* < 0.05 vs. with IS.

NF-κB/MAPK is a major signaling pathway for inflammatory cytokine production in various cell types. IS induced the phosphorylation of extracellular signal-related kinase p38, JNK, but not ERK1/2 in macrophages (Figure 4A–F). IS also induced phosphorylation of NF-κB p65 in macrophages (Figure 4G,H). BAY11-7082 (BAY), an inhibitor of NF-κB, strongly reduced the IS-induced increase of pro-IL-1β (Figure 5). Thus, IS induced the activation of macrophages with MAPK and NF-κB signaling pathways, which were associated with pro-IL-1 expression.

### 2.3. IS Did Not Activate the NLRP3 Inflammasome in Macrophages

**Figure 6:**
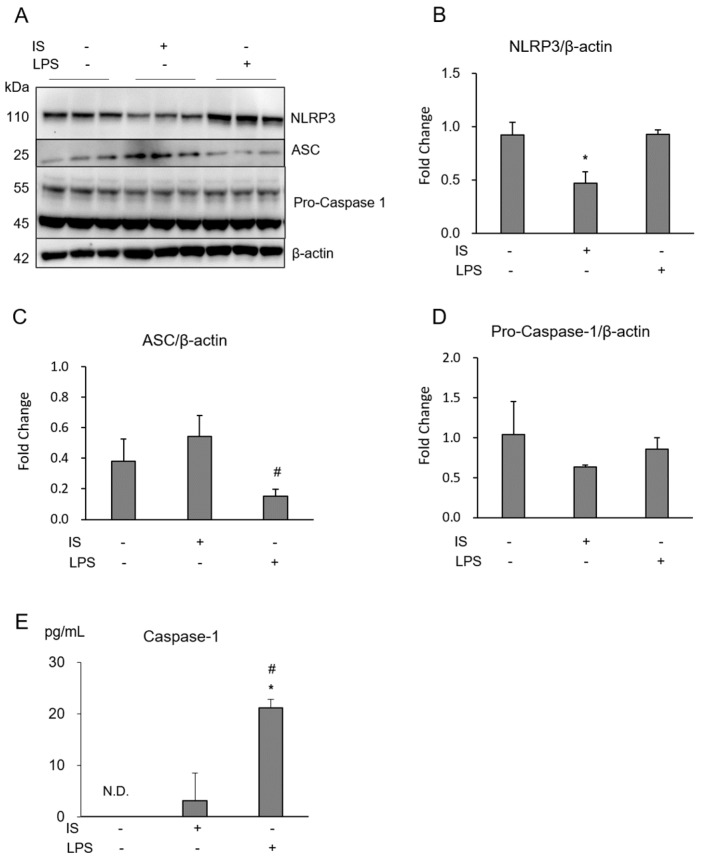
NLRP3 inflammasome activity in THP-1 macrophages exposed to IS. Effect of 1 mM (213 μg/mL) IS and 100 ng/mL LPS on intracellular NLRP3 (**A**,**B**), ASC (**A**,**C**), and pro-caspase 1 (**A**,**D**) were determined by Western blotting. Activation of caspase-1 in THP-1 macrophages exposed to IS or LPS were quantified by ELISA to detect proteins secreted into the cell culture supernatant (**E**). Data represent the mean ± SD of three experiments. * *p* < 0.05 vs. without IS and LPS, and # *p* < 0.05 vs. with IS.

The NLRP3 inflammasome is an essential pathway for the host immune response, which leads to the maturation of IL-1 and the activation of caspase-1. IS decreased macrophage NLRP3 protein expression (Figure 6A,B), but not ASC and pro-caspase 1 (Figure 6A,C,D). Caspase-1 was not activated with IS, while LPS increased it significantly (Figure 6E). These results indicate that IS did not activate the NLRP3 inflammasome.

## 3. Discussion

In this study, we demonstrated that IS induced macrophage inflammatory reactions by activating AhR, NF-κB, and MAPK, but the NLRP3 inflammasome was not activated. Accumulation of uremic toxins including IS is a CKD-specific risk factor that accelerates atherosclerosis. We previously showed that IS deposits in CKD-induced acceleration of atherosclerotic lesions [5] and reacts to macrophages directly, inducing inflammation and abnormal lipid handling [8], but the detailed mechanism is not completely understood.

IS induces inflammation in various types of cells, and several reports have demonstrated the importance of interactions with AhR. The AhR is a nuclear transcription factor that mediates toxic responses by adjusting numbers of metabolism-related enzymes, including CYP1A1. The AhR binds to the AhR nuclear translocator (ARNT) and the ligand-bound AhR/ARNT complex translocates from the cytoplasm into the nucleus to modulate the expression of target genes, such as CYP1A1 and CYP1B1 [14]. IS inhibited the expression of fetuin-A through AhR activation in a human hepatoma HepG2 cell line [15]. IS downregulates the expression of Mas receptor, which is associated with the inhibition of the renin–angiotensin system, via the organic anion transporter 3/AhR/signal transducer and activator of transcription 3 pathway in proximal tubular cells [11]. In human umbilical vein endothelial cells (HUVECs), AhR also mediates IS-induced cellular senescence [16] and monocyte chemoattractant protein-1 expression [17]. Furthermore, IS upregulates several AhR-related genes, which are associated with tissue factor production in HUVECs as well as peripheral blood mononuclear cells [18]. In contrast, few studies have assessed the role of AhR in macrophages. Hypoxia-inducible factor knockout macrophages have shown increased expression of CYP1A1 and reduced AhR nuclear translocator [19]. Polycyclic aromatic hydrocarbons have been found to inhibit the differentiation of human monocytes into macrophages through the AhR pathway [20]. This is the first study in which the role of AhR in macrophages reacted with uremic toxins in vitro is assessed and was conducted to understand the mechanism of uremic toxin-induced acceleration of macrophage inflammation. Based on our previous findings [8] and on other reports [21,22], we chose macrophages differentiated from THP-1 cells and 1 mM IS. The dose of IS (1 mM, 213 μg/mL) was higher compared to the circulating level as protein-unbound form in kidney disease patients, but the inflammatory reaction of THP-1 macrophage showed maximum when cells were exposed with 1 mM IS [8]. It is possible that the concentration of IS in the atherosclerotic lesion may be much higher than the circulating level, or macrophages are accelerated inflammatory reaction with not only IS, but various uremic toxins in vivo. In this study, IS interacted with AhR during IL-1 production (Figure 1 and Figure 2) and appears to be a first step in the interaction of IS with macrophages, as in other types of cells. AhR reacted with agonists forms heterodimer with Arnt. The heterodimer binds to the xenobiotic responsive element, which induces the expression of target genes including CYP1A1 and CYP1B1 [23]. CYP1A1 is the most potent inducer of the reaction of cells to 2,3,7,8-tetrachlorodibenzo-p-dioxin [24]. Our study showed an enhancement in the macrophage classical pathway of AhR, which includes CYP1A1 and CYP1B1, and AhRR expression with IS, as found in previous reports on endothelial cells [17]. In this study, the proteasome inhibitor MG132 recovered the IS-induced reduction of AhR expression, but did not alter pro-IL-1 expression (Figure 3). Inhibition of the AhR proteasome may not effectively suppress IS-related interactions with AhR, and the reaction with AhR is not the only pathway for the IS reaction in macrophages. Further studies are needed to understand the detailed mechanism of the reaction of IS with macrophages.

It is well-known that macrophage inflammation and apoptosis are enhanced by MAPK and NF-activation. For example, in vitro, LPS enhances the phosphorylation of mouse macrophage NF-κ p65, Ik-, ERK, JNK, and p38, which was inhibited by SC-E3, a novel herbal formula [25]. Macrophage-specific NF-κB knockout in apolipoprotein E-deficient mice reduced the high-fat-diet-induced acceleration of atherosclerosis [26]. In contrast, IS activated NF-κB/MAPK cascades, which enhanced inflammatory reactions in several cell types. In human umbilical vein endothelial cells, IS increases the expression of intercellular adhesion molecule-1 and monocyte chemotactic protein-1 and enhances reactive oxygen species production and NF-κ activation [21]. In proximal tubular cells, IS activates p53 expression, which induces cellular senescence and fibrosis [27]. As predicted in previous studies, we demonstrated that IS increased the phosphorylation of NF-κB p65 and MAPK (p38 and JNK) in macrophages, which may be the main pathway especially for macrophage inflammation (Figure 4). Inhibition of NF-κB with BAY11-7082 suppressed IS-induced pro-IL-1 expression in macrophages (Figure 5), which may be an attractive therapeutic option for preventing uremic toxin-induced macrophage inflammatory reactions.

Inflammasomes are multi-protein oligomers in the cytoplasm that intensify inflammation in response to a wide range of danger signals, including pathogen-associated molecular patterns and danger-associated molecular patterns [13,28]. The inflammasome consists of NLRP, ASC, and pro-caspase 1 which activate caspase-1 to cleave pro-IL-1. Thus, the NLRP3 inflammasome is thought to be important in the acute inflammatory response in cells. However, in this study, IS did not promote activation of the macrophage NLRP3 inflammasome (Figure 6), but strongly activated AhR-NF-κB/MAPK cascades (Figure 2 and Figure 4). Many previous studies reported that activation of the NLRP3 inflammasome in macrophages induces cellular inflammation, apoptosis, and cell death in atherosclerotic lesions [13]. Our results showed that IS decreased NLRP3, but not the expression of ASC, pro-caspase 1, or caspase 1 activity (Figure 6). The decreases in NLRP3 with IS and in ASC and pro-caspase 1 with LPS may be due to the consumption of inflammasome. Caspase 1 was not activated with IS in this experimental setting (Figure 6).

In patients with kidney disease, various types of uremic toxins accumulate in the blood vessels and induce chronic inflammation, leading to acceleration of atherosclerosis. Some IS-inducing inflammation in macrophages results from the acceleration of AhR-NF-κB/MAPK cascades; however, non-activation of the NLRP3 inflammasome with IS may restrict IL-1β maturation. This reaction may be explained by chronic inflammation in CKD-induced atherosclerotic lesions. Further studies using other uremic toxins for other atherosclerosis-associated cells and the inflammasome in macrophages from CKD patients are needed to understand the detailed mechanism of CKD-associated CVD, since not only uremic toxins but also several CKD-associated factors can induce phenotypic changes in macrophages in atherosclerotic lesions.

## 4. Conclusions

IS induced macrophage inflammation by activating AhR-NF-κB/MAPK cascades, but did not activate the NLRP3 inflammasome in vitro, which may explain the sustained chronic inflammation in CKD patients.

## 5. Materials and Methods

### 5.1. Cell Culture

THP-1 human monocytic leukemia cells (American Type Culture Collection, Manassas, VA, USA) were cultured in RPMI 1640 (Gibco-BRL, Grand Island, NY, USA), supplemented with 10% heat-inactivated fetal bovine serum (Gibco), 100 μg/mL penicillin, 100 μg/mL streptomycin, 10 mM HEPES, 1×MEM vitamin, and 0.5 µM 2-mercaptoethanol (Gibco). Cells were incubated at 37 °C in humidified air with 5% CO_2_. THP-1 cells at a density of 1 × 10^6^/mL were differentiated into macrophages using 50 ng/mL phorbol 12-myristate 13-acetate (Sigma-Aldrich, St. Louis, MO, USA) for 72 h (THP-1 macrophages). Macrophage differentiation from monocytes was detected by their adherence to the culture plate.

### 5.2. Measurement of Inflammatory Cytokines in Medium

Macrophages were exposed to IS at a concentration of 1.0 mM (213 μg/mL) or lipopolysaccharide (LPS) at a concentration of 100 ng/mL in fetal bovine serum-free medium for 24 h. Cell supernatants were collected, and the concentrations of IL-1β and caspase-1 were measured using human IL-1β (GEN-PROBE, San Diego, CA, USA) and human caspase-1 ELISA Kits (R&D Systems, Minneapolis, MN, USA) according to the manufacturer’s protocols.

### 5.3. Measurement of mRNA Expression

Macrophages were exposed to 1.0 mM IS or 100 ng/mL LPS for 24 h. Total RNA was extracted from cells using the GenElute mammalian total RNA miniprep kit (Sigma-Aldrich) in accordance with the manufacturer’s instructions. Quantitative real-time PCR was performed using the one-step SYBR plus RT PCR kit on a thermal cycler dice real-time system (TP900, Takara, Shiga, Japan). Primers for human pro-IL-1β (HA106116, Takara), AhR (HA189225, Takara), CYP1A1 (HP200471, ORIGENE, Rockville, MD, USA), CYP1B1 (HA278296), AhRR (HA103369), Arnt (HA124219), NLRP3 (HA222280, Takara), and GAPDH (HA067812, Takara) were used to quantify mRNA expression. GAPDH was used as an internal control.

### 5.4. Detection of Proteins in Cells

THP-1 macrophages were exposed to 1.0 mM IS for 24 h. Western blot analysis was performed to detect the expression of pro-IL-1β, ASC, NLRP3, and AhR and detect the phosphorylation of ERK1/2, p38, JNK, and NF-κB p65 in cells. Whole-cell lysates were prepared in a RIPA buffer, which is composed of 25 mM Tris-HCl (pH 7.5), 150 mM NaCl, 1% NP-40, 1% sodium deoxycholate, and 0.1% sodium deoxycholate. One tablet of complete protease inhibitor cocktail (Roche, Basel, Switzerland) and 1 tablet of phosphatase inhibitor (Roche) were added to 10 mL of RIPA buffer before use. The RIPA lysis cell lysates were homogenized by passing through a 25-gauge needle 15 times. After centrifugation with 15,000 rpm at 4 °C for 30 min, the supernatants of lysates were separated and used as protein solutions. The protein (20 µg) was separated on NuPAGE 4–12% SDS-polyacrylamide gels (Life Technologies, Carlsbad, CA, USA) and transferred to a polyvinylidene fluoride membrane (Life Technologies). Membranes were blocked with 5% nonfat dry milk in Tris-buffered saline containing Tween 20. To detect pro-IL-1β, ASC, NLRP3, AhR, MAPK, and NF-κB p65, membranes were incubated overnight at 4 °C with primary antibodies. Horseradish peroxidase-linked secondary antibodies, goat anti-mouse IgG-HRP conjugated and goat anti-rabbit IgG-HRP conjugated antibodies, were used, and signals were visualized by chemiluminescence (Lumi Vision PRO LPR-45/NP-1, TAITEC, Saitama, Japan). Chemiluminescence intensity was calculated using ImageJ software (NIH, Bethesda, MD, USA). Ah receptor (H-211) rabbit polyclonal IgG (diluted 1:5000), ASC (N-15)-R rabbit polyclonal IgG (diluted 1:250), IL-1β (H-153) rabbit polyclonal IgG (diluted 1:200), goat anti-mouse IgG-HRP conjugated (1:8000), and goat anti-rabbit IgG-HRP conjugated (1:8000) were purchased from Santa Cruz Biotechnology (Dallas, TX, USA). NF-κB p65 (C22B4) (diluted 1:1000), P-NF-κB p65 (S536) (93H1) rabbit mAb (diluted 1:1000), NLRP3 (D4D8T) rabbit mAb (diluted 1:300), p44/42 MAPK (Erk1/2) (137F5) rabbit mAb (diluted 1:1000), P-p44/42 MAPK (T202/Y204) (D13, 14, 4E) XP (R) rabbit mAb (diluted 1:2000), p38 MAPK (D13E1) XP (R) rabbit mAb (diluted 1:1000), P-p38 MAPK (T100/Y182) (D3F9) XP (R) rabbit mAb (diluted 1:1000), SAPK/JNK rabbit Ab (diluted 1:1000), and P-SAPK/JNK (T183/Y185) (81E11) rabbit mAb (diluted 1:1000) were purchased from CST Japan (Tokyo, Japan). Anti-β-ActinpAb-HRP-Direct rabbit Ig (aff.) (diluted 1:8000) was purchased from MBL (Nagoya, Aichi, Japan).

### 5.5. Reaction with NF-κB Inhibitor and Protease Inhibitor to Macrophages

THP-1 macrophages were exposed to the NF-κB inhibitor 10 M BAY11-7082 (Sigma-Aldrich) 1 h before IS addition as described previously [29]. After the 24 h reaction, whole cell lysates were collected using RIPA buffer. The proteasome inhibitor MG132 (10 M) (Sigma-Aldrich) was added to THP-1 macrophages 1 h before the reaction with IS.

### 5.6. Statistics

The results are expressed as the mean ± standard deviation. Statistical differences were assessed using an unpaired Student *t*-test or a single-factor analysis of variance followed by Bonferroni correction. *p* < 0.05 was considered significant.
